# Ultrafast inactivation of SARS-CoV-2 by 254-nm UV-C irradiation on porous and non-porous media of medical interest using an omnidirectional chamber

**DOI:** 10.1038/s41598-023-39439-1

**Published:** 2023-08-04

**Authors:** Bertrand Maubert, Camille Theillière, Prescillia Jany, Thomas Bourlet, Jérôme Deschamps, Bruno Pozzetto, Fateh Singh, Emilie Gadea

**Affiliations:** 1 Laboratoire de Biologie, Centre Hospitalier Emile Roux, 43000 Le Puy en Velay, France; 2 Unité de Recherche Clinique, Centre Hospitalier Emile Roux, 43000 Le Puy en Velay, France; 3 CIRI, Centre International de Recherche en Infectiologie, GIMAP Team, Univ St-Etienne, INSERM U1111, CNRS UMR5308, ENS de Lyon, UCBL1, Univ Lyon, 42023 Saint-Etienne, France; 4 Service des Agents Infectieux, Centre Hospitalier Universitaire de Saint-Étienne, 42055 Saint-Etienne, France; 5 ON-LIGHT SAS, SMO Biopole Clermont-Limagne, 63360 Saint Beauzire, France; 6 U1059, Equipe DVH, Mines Saint-Etienne, Univ Lyon, Univ St-Etienne, 42000 Saint-Etienne, France

**Keywords:** SARS-CoV-2, Antiviral agents, Occupational health

## Abstract

Covid-19 has spurred a renewed interest in decontamination techniques for air, objects and surfaces. Beginning in 2020, urgent effort was done to permit the reuse of UV-C for inactivating SARS-CoV-2. However, those studies diverged widely on the dose necessary to reach this goal; until today, the real value of the sensitivity of the virus to a 254-nm illumination is not known precisely. In this study, decontamination was performed in an original UV-C large decontamination chamber (UVCab, ON-LIGHT, France) delivering an omnidirectional irradiation with an average dose of 50 mJ/cm^2^ in 60 s. Viral inactivation was checked by both cell culture and PCR test. SARS-CoV-2 was inactivated by UV-C light within 3 s on both porous (disposable gown) and non-porous (stainless steel and apron) surfaces. For the porous surface, an irradiation of 5 min was needed to achieve a completely negative PCR signal. The Z value estimating the sensitivity of SARS-CoV-2 to UV-C in the experimental conditions of our cabinet was shown to be > 0.5820 m^2^/J. These results illustrate the ability of this apparatus to inactivate rapidly and definitively high loads of SARS-CoV-2 deposited on porous or non-porous supports and opens new perspectives on material decontamination using UV-C.

## Introduction

Following the emergence of SARS-CoV-2 virus at the beginning of 2020, the spread of Covid-19 has taken most health organizations by surprise, which resulted notably in an acute shortage of personal protective equipment (PPE)^1,2^, including masks, gowns, gloves and respirators, together with a rush to sanitize all surfaces and objects that may have been in contact with the virus^3^. Different disinfecting solutions have been explored, notably ozone^4^, gamma irradiation^5^, hydrogen peroxide^6,7^, heat treatment^7^ and quaternary ammonium salts^8^. UV-C is a well-known technology that has existed for more than 150 years and can rapidly kill viruses and bacteria in an environmentally-friendly way, without the need for chemicals.

Studies using UV-C for inactivating SARS-CoV-2 started as soon as the beginning of 2020, when the shortages were the most acute, mainly focused on masks and respirators^7,9^ and whole room decontamination^10,11^, with the aim to demonstrate virus elimination as determined by PCR, even if this technique does not evaluate the infectivity of the virus but rather detects the presence of viral genomic material in the sample. More recently, studies have looked at the viral sensitivity of SARS-CoV-2 to 254-nm light^12–14^ produced by low pressure mercury lamps. A past study on the same viral subgenus (*Beta-Coronavirus*) had shown great heterogeneity of results^15^, mainly on non-porous substrates (Petri dishes or glass plates).

The present study considers inactivation of SARS-CoV-2 on both non-porous surfaces (stainless steel, plastic apron) and a fibrous, porous surface (gown) for a range of UV-C doses using a large decontamination chamber equipped of high-power mercury lamps emitting omnidirectionally at a wavelength of 253.7 nm and delivering an average dose of 50 mJ/cm^2^ in 60 s, on each side, for opaque items positioned vertically at the centre of the cabinet. In all cases, inactivation was checked by PCR and viral culture. Moreover, for the porous surface, the kinetics of the PCR signal was determined with respect to UV-C dose. The apparent Z value estimating the sensitivity of SARS-CoV-2 to UV-C in the experimental conditions of our cabinet was also calculated.

## Methods

### UV irradiation with UVCab

UVCab is a UV-C large decontamination chamber (60 cm × 60 cm × 100 cm) developed by ON-LIGHT, France. The interior is covered with highly reflective aluminium on all sides, with specific optical design to ensure maximum irradiation intensity and uniformity on the treatment zone. UVCab uses high power mercury lamps emitting at a wavelength of 253.7 nm. The average lighting intensity provided by the device is 8.33 W/m^2^ (on each side of the central vertical plane), measured with a HD2102 radiometer (DeltaOhm, Italy) with a LP471UVC cosine corrected probe (DeltaOhm, Italy). This corresponds to an average dose of 50 mJ/cm^2^ in 60 s, on each side, for opaque items positioned vertically at the centre. The irradiation is omnidirectional to limit shadowing. The device is equipped with a safety system that locks the door until the end of its cycle. The decontamination cycle time depends on the material of the equipment to be decontaminated (partly transparent or opaque), the number of layers that compose it and the nature of the material. During all experiments, samples were irradiated in the same conditions (with a plastic holder leaving a 5 × 5 cm exposed window open on both sides) with the same angle of exposure with respect to the source (in vertical position, parallel to the UV-C sources). Only the stainless-steel samples were tested both in the vertical and horizontal position to evaluate the impact of the exposure angle on the UV-C effectiveness.

### Cell culture and viral strain

Vero E6 cells (ATCC CRL-1586) were used to confirm the viral inactivation. Cells were maintained in high glucose Dulbecco’s Modified Eagle Medium (DMEM, Sigma) supplemented with 2% of foetal bovine serum (FBS) and antibiotics (Penicillin/Streptomycin). They were inoculated into 24-well plates with a volume of 500 µl and a density of 1 × 10^5^ cells per well and incubated at 37 °C and 5% CO_2_ until use.

The Alpha strain (20I/501Y B.1.1.7 lineage) of SARS-CoV-2 that was used in all the experiments was sequenced and deposited at GISAID (https://www.gisaid.org/) (accession number: EPI_ISL_1707039). The infectious viral titre, determined with the Reed and Muench method^16^, was expressed in TCID_50_. The virus stock was used at the concentration of 10^5,5^ TCID_50_/150 µl, which corresponds to the average viral concentration that can be found in a patient recently infected by SARS-CoV-2^17,18^.

### Design of the study and experimental protocol

All the experiments using infectious material were performed in a biosafety level 3 laboratory. Three different surfaces were selected to assess the virus inactivation ability of UV-C: semi-transparent porous cloth (disposable non-spun 20 g/m^2^ gowns, ref 938481, Prop, France), semi-transparent non porous plastic (aprons, ref: 161105, Euromedis, France) and opaque non-porous stainless-steel holders (ref: Inox 316L, Cellux, France). For the semi-transparent materials, the transmission ratio at 254 nm was determined to be 36.0% and 15.2% for gown and apron, respectively.

A volume of 100 µl of an aliquot fraction of the viral stock, diluted 1:10, was inoculated onto the 15 samples of each tested surface, with 20 min drying time under a biosafety cabinet. Cloth and plastic samples were prepared by cutting 7 × 7 cm swatches. Stainless steel samples measured 3 cm on each side. The 15 samples of each category were treated in triplicate using five different conditions: a range of 4 irradiation times were tested (3, 8, 15 and 30 s corresponding to a dose on the contaminated surface of 2.5, 6.7, 12.5 and 25.0 mJ/cm^2^ for stainless-steel samples, 3.4, 9.11, 17 and 34 mJ/cm^2^ for gown samples and 2.88, 7.72, 14.4 and 28.8 mJ/cm^2^ for apron samples) and compared to the result of non-irradiated control samples that exhibited exactly the same treatment except irradiation. After treatment, each sample was introduced into 120 ml-plastic jars and suspended in 5 ml of culture medium (DMEM) before being vortexed for 30 s. After addition of 5 ml of culture medium to completely cover the sample, each jar was vortexed again for 30 s and the supernatants were collected for cell culture.

For cell culture experiments, the medium was removed from each well of the 24-well microplates covered with Vero cells and 250 µl of each sample or control medium was added. After a contact phase of 15 min at 37 °C under 5% CO_2_, 350 µl per well of DMEM were added for a final volume of 600 µl and the plates were incubated for 5 days under the same conditions. Results were observed microscopically for recording of a characteristic cytopathic effect. In parallel, a 200 µl aliquot fraction of each supernatant was suspended in lysis buffer for PCR experiments.

The real-time PCR technique that was used targets two regions located in the nucleocapsid and RNA-dependent polymerase genes of SARS-CoV-2 (SARS-CoV-2 r-GENE®, bioMérieux, France); it was performed on Applied Biosystem 7500 Fast (ThermoFisher, France) after extraction of nucleic acids on the NucliSENS® easyMAG® platform (bioMérieux) according to the recommendations of the manufacturer.

### Apparent Z value estimation for surfaces contaminated by SARS-CoV-2

Pathogens are inactivated when a photon, due to it’s energy, is able to create pyrimidine photoproducts that prevent translation and/or translation^19^.

The sensitivity of any virus exposed to inactivation by UV-C can be succinctly estimated by its apparent Z value representing the virus sensitivity constant when exposed to the 254-nm mercury ray. Thus, for a given agent, its Z value is able to accurately predict how the virus will behave when exposed to a given dose of UV-C^20^. To calculate this value, it is needed to determine the irradiation dose required to eliminate 90% of the microorganism on an exposed surface (D_90_) by using the following formula:

1 $${\text{D}}_{{{9}0}} = \, - {\text{ln}}\left( {0.{1}} \right)/{\text{Z}}.$$

This formula can be extended to other elimination ratios:

2 $${\mathrm{D}}_{\mathrm{x}}=-\frac{\mathrm{ln}\left(1-{\text{x}}/{100}\right)}{\mathrm{Z}}.$$

## Results

### Inactivation of SARS-CoV-2 on non-porous surfaces

Data obtained on non-porous surfaces (apron or stainless-steel supports) showed an efficiency of UV-C on the inactivation of viral replication using an infectious concentration of 1.5 × 10^5.5^ 50% tissue culture infective doses (TCID_50_) per150 µl, from 3 s of irradiation (2.5 mJ/cm^2^ for stainless-steel, 3.4 mJ/cm^2^ for apron). Indeed, in cell culture, no cytopathic effect was observed regardless of the irradiation time applied to the two supports.

**Figure 1:**
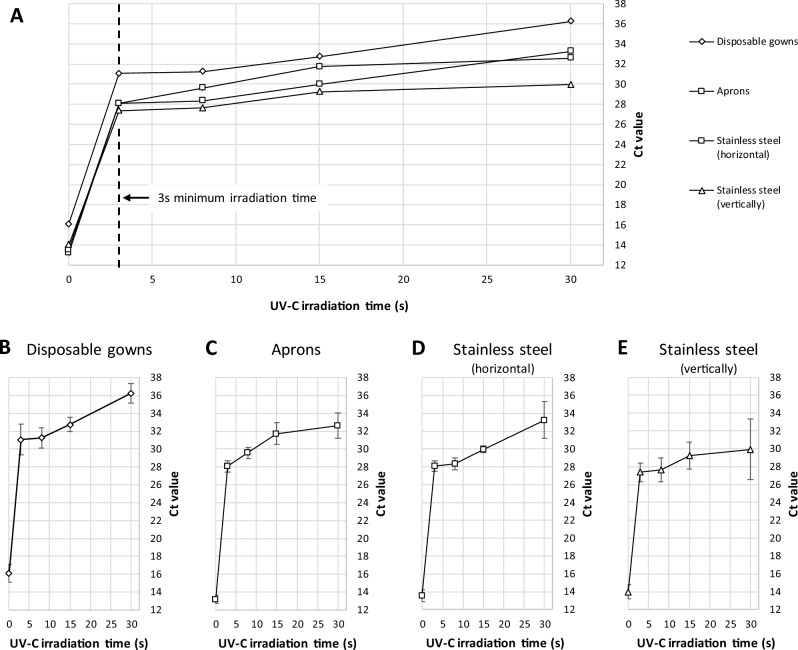
Inactivation of SARS-CoV-2 after exposure to different UV-C doses. Representation of viral inactivation by PCR technique on different surfaces (gown, apron, stainless steel) subjected to increasing doses of UV-C irradiation. (**A**) All surfaces, (**B**) Disposable gown, (**C**) Aprons, (**D**) Stainless steel (horizontal), (**E**) Stainless steel (vertical). Error bars show standard deviation.

The supernatants were also tested with PCR technique: a rise in cycle threshold (C_T_) value is representative of a decrease in concentration of viral genomic material sensitive to the primers. For the apron samples, C_T_ ranged from 28.1 to 32.6 for irradiation times of 3 to 30 s respectively (Fig. 1). For the stainless-steel supports placed in vertical position in the cabinet, the C_T_ values varied from 28.4 to 33.2 for irradiation times of 3 to 30 s respectively. From similar samples exposed in a horizontal position to the UV-C source, the C_T_ values varied from 27.4 to 29.9 for irradiation times of 3 to 30 s respectively (Fig. 1), which demonstrates that the angle of exposure of the supports with respect to the UV-C source has no impact on the irradiation efficiency. In addition, the latter experiment with stainless-steel samples placed in horizontal position was performed a second time to test its reproducibility: the results obtained for the two series were almost identical (data not shown). It should be noted that the standard deviation for the longest irradiation time is larger than for other results, indicating a possible issue with a single sample.

### Inactivation of SARS-CoV-2 on porous surfaces

Concerning the disposable gowns samples, the results reported in cell culture and in PCR are close to those reported for the non-porous surfaces. In cell culture, no cytopathic effect was observed even with short irradiation duration of 3 s (2.5 mJ/cm^2^) on an infectious viral concentration of 1.5 × 10^5.5^ TCID_50_/150 µl, suggesting that UV-C is highly effective in inactivating viral replication on fibrous, porous surfaces. Tested by PCR technique, the supernatants exhibited C_T_ values ranging from 31.0 to 36.2, revealing the presence of residual viral RNA unable to infect cells in culture. The C_T_ values increased linearly with respect to the applied irradiation dose (Fig. 1).

To study the kinetics of viral RNA degradation, a complementary test was performed on porous material (disposable gown) to determine the irradiation time for which the viral RNA is no more detected by PCR. Extended times were used up to 30 min (Table 1). The results show that an irradiation time of 30 s lead to a C_T_ value over 35; 1 min-irradiation leads to a C_T_ value over 40 and at 5 min no PCR signal was recorded, which indicates that dimerization has occurred in the attachment zone of primers.

**Table 1:** Kinetics of PCR signal inactivation on disposable gowns.

	Cycle threshold (C_T_) value		
	Nucleocapsid gene	RNA-dependent polymerase gene	
No irradiation*	14.1	13.6	
Irradiation of 3 s	29.4	28.8	Minimum practical irradiation time
Irradiation of 8 s	31.1	30.9	
Irradiation of 15 s	32.3	32.4	
Irradiation of 30 s	36.6	39.9	
Irradiation of 1 min	40.4	*ND*	
Irradiation of 5 min	*ND*	*ND*	
Irradiation of 10 min	*ND*	*ND*	
Irradiation of 15 min	*ND*	*ND*	
Irradiation of 30 min	*ND*	*ND*	

Samples were tested with different irradiation times in order to determine the time necessary to negative the PCR signal, signifying total degradation of the viral genetic material.

*ND* not detected.

*This sample was the only one that gave a cytopathic effect when inoculated on cell culture.

### Estimation of apparent Z value for surfaces contaminated by SARS-CoV-2

In this study, a boundary of the apparent Z value was calculated thanks to the data obtained during the titration of the variant and using Eq. (1) described in the “Methods” section. From the titration, a dilution of 10^6.32^ resulted in a reduction of 50% of infectivity. On stainless steel, an irradiation of 25 J/m^2^ was sufficient to inactivate the virus, which results in a more than a 10^6.32^ reduction. Consequently, the apparent Z value is > 0.5820 m^2^/J, which corresponds to a maximum D_90_ value of 3.9563 J/m^2^.

## Discussion

In order to correctly design and operate UV-C decontamination apparatuses with maximum efficacy, it is important to know the key parameters that govern viral inactivation. The effectiveness of UV-C decontamination depends on the pathogens to be inactivated, the doses of irradiation applied, the characteristics of the support materials, the experimental conditions (in particular the absorption of the medium) and the environmental context (temperature, humidity). If some of these parameters are easy to control (the dose for example), others are linked to operating conditions. Knowing the sensitivity of the pathogen on different substrates is therefore a key element for reducing uncertainties in operations.

Data present in the literature indicate that the nature of the phase of the viral sample (solid surface, liquid medium) has a huge influence on the irradiation efficacy, notably by the absorption of a part of the UV-C radiation^15,21,22^ in the suspension medium or by pollution by foetal bovine serum (FBS) or other compounds. For instance, Biasin et al.^4^ showed that the presence of a 1 mm-layer of culture medium with transmission factor of 0.68 reduces the illumination from 5.4 mJ/cm^2^ at the top interface to 3.7 mJ/cm^2^ at the bottom of the quartz cuvette. Higher doses of irradiation are then necessary to reach the same level of efficiency. By contrast, drying the viral suspension for a short time on steel or polymers does not reduce infectivity^23^, which ensures the validity of dried assays.

In the present study, 10% of FBS was added to the culture medium for growing the cells, which means that some residual absorption could be expected, impacting decontamination time and UV sensitivity calculation. It would also be interesting to measure UV-C absorption of biological fluids like saliva, bronchoalveolar fluid, blood or semen, in order to extrapolate the practical decontamination to be expected in field use.

Porous materials are especially challenging. Indeed, surface rugosity of porous materials can potentially reduce effectiveness by shielding pathogens from UV-C rays^24,25^. A careful examination of the material to be treated has to be carried. This has mostly been done for N95 respirators^26–28^. The present study has carried tests on 3 materials, but they represent a very small subset of the available materials, leaving further research open.

Non porous, flat, hard surfaces with dried virus solution, such as stainless-steel, with minimal pollution, can be seen as a “best case”, requiring the least amount of energy to successfully inactivate the pathogen. However, the tests carried on the other two polymer materials, both porous and non-porous, showed a good inactivation profile. Additional research on lower doses or on single side illumination would help refine the knowledge of inactivation dose needed for SARS-CoV-2.

In a clinical setting, a large over-irradiation is to be preferred. A homogenous, omnidirectional illumination is preferable, as the precise orientation of the target is rarely known. This allows some shadowing, some humidity and potentially some surface contamination in day-to-day operations. This over-irradiation is also beneficial in terms of efficiency check in clinical settings, where cell culture is unpractical. A low irradiation dose would leave un-dimerized primers attachment zones and a false positive PCR signal since this technique cannot discriminate between live and non-infectious virions^29^. Our results obtained in viral culture and correlated with those of the PCR technique show that, at irradiation times lower than 30 s, viral RNA is not 100% degraded by UV-C since PCR signals are still detectable. Nevertheless, the data from viral culture prove that the virus is well inactivated. On the gown sample, a minimum of 1 min treatment was shown to be necessary for generating no signal in PCR. However, a minimal irradiation time of 3 s, and enabling reduction of over 6Log_10_ of the viral load, seems adequate for the decontamination of most objects.

The research was conducted using Alpha strain. Results are expected to be equivalent with other strains.

**Figure 2:**
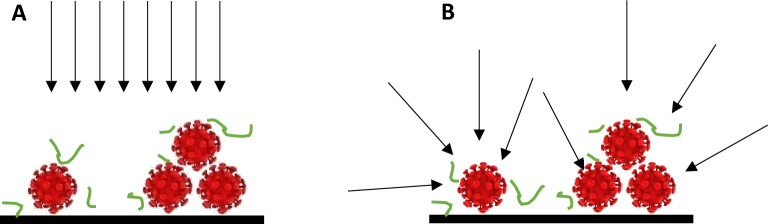
Illustration of the effect of planar vs omnidirectional illumination of dried virus solution. Black: substrate; Red: SARS-CoV-2 virions; Green: vestigial proteins from culture media. (**A**) Planar illumination leads to shadowing by proteins and virions. (**B**) Omnidirectional illumination eliminates shadowing.

In the present study, the apparent UV-C sensitivity was shown to be much higher than previously reported (Table 2), with Z value > 0.5820 m^2^/J. This value is at least 3 times higher than reported by Biasin et al.^4^, Storm et al.^9^, Ma et al.^14^ and Martínez-Antón et al.^21^. Experimental conditions of these studies are very different, as they are all using a nearly planar illumination: Storm et al. used a collimated beam; Biasin et al. also used an aperture to make a “spatial filter”, limiting the half angle of input rays to 30°; Martínez-Antón et al. restricted the lamp length to a 5 mm-window placed 36 cm away from the target, achieving very low angular spread and a nearly planar illumination. In contrast, the UVCab apparatus has an optical design made to have an omnidirectional illumination of the target (Fig. 2). This is illustrated by stainless-steel samples that have been tested both in the vertical and horizontal directions, with very close PCR values, indicating low dose deviation between those two extreme positions (Fig. 1). In this configuration, the dose received on the surface cannot be described in terms of planar irradiance and the concept of spherical irradiance must be preferred. A good explanation of the differences between planar and spherical irradiance was given by Ashdown et al.^30^. The concept of spherical irradiance is also to be found in the much higher susceptibility of pathogens in the aerosol form, as shown by Kowalski et al.^31^.

**Table 2:** Apparent Z values for SARS-CoV-2 after UV-C irradiation reported from different studies.

Apparent Z value at 254 nm (m^2^/J)	D_90_ J/m^2^	Reference
0.1867	13.33^#^	Biasin et al.^4^
0.0921	25^+^	Storm et al.^9^
0.20536	11.21^+^	Storm et al.^9^
0.177*	13	Ma et al.^14^
0.21	10.8	Martínez-Antón et al.^21^
> 0.582	3.96	This study

^#^Calculated from log_3_ inactivation.

^+^Calculated from Z.

*Calculated from D_90_.

We expect results to be similar for all SARS-CoV-2 variants (Table 3), as UV-C have a very specific mode of action. The UV-C photon is sufficiently energetic to create pyrimidine, which prevent transcription or replication. Kowalski et al. have established a genomic model to determine the susceptibility of different pathogens. The sensitivity of different strains of SARS-CoV-2 being structurally very close to each other, we can estimate the difference of sensitivity based on these occurrences.

**Table 3:** Pyrimidine, and pyrimidine sequences in some selected SARS-CoV-2 variants.

			Pyrimidines in genome	No of pyrimidine doublets	No of purine/pyrimidine/pyrimidine
	SARS-CoV-2/human/USA/USA-WA1/2020	GenBank: MT246667.1	15,086	7597	7676
Alpha	SARS-CoV-2/human/USA/GA-CDC-4201216-001/2021	GenBank: 0K045638.1	15,071	7590	7668
	*Difference to USA-WA1*		*0.10%*	*0.09%*	*0.10%*
Delta	SARS-CoV-2/human/Colombia/CO-SAN-UW21120626151/2021	GenBank: 0N150700. 1	15,014	7554	7601
	*Difference to USA-WA1*		*0.48%*	*0.57%*	*0.98%*
	*Difference to Alpha*		*0.38%*	*0.47%*	*0.87%*
XBB.1.16	SARS-CoV-2/human/THA/MTM_01_682/2023	GenBank: 0Q946922.1	14,950	7519	7612
	*Difference to USA-WA1*		0*.90%*	*1.03%*	*0.83%*
	*Difference to Alpha*		*0.80%*	*0.94%*	*0.73%*

As it can be seen, there is less than 1% between an Alpha strain and other strains, even with very recent genomes like XBB.1.16.

It is therefore highly likely that the sensibility of other future strains will be very close to that of past variants, unless a large genomic event occurs (large deletion or duplication).

In conclusion, 254-nm UV-C radiation has demonstrated to be very efficient in SARS-CoV-2 inactivation and proves once again to be an effective process that can be used in many cases such as surface decontamination or air purification in confined spaces. The use of a specifically designed device such as the UV-Cab device can easily be applied in order to limit the transmission of viruses and bacteria by contact, as it is capable of ultrafast virus inactivation (3 s) on porous or non-porous media as shown on SARS-CoV-2. The Z value obtained in this study is almost 3 times higher than commonly observed by others, which resulted in approximately 3 times less time for inactivating the virus. That is to be explained by the specific design of the inner of UVCab that permits an omnidirectional illumination of the objects to be decontaminated. This kind of apparatus could be applied to the decontamination of different medical devices such as, for instance, cell phones used in hospitals that have been shown to constitute a significant source of nosocomial infections^32^.

### Supplementary Information

Supplementary Table S1.

## Data Availability

The main data are presented in the manuscript. Additional data are available under request to the corresponding author. The Alpha strain (20I/501Y B.1.1.7 lineage) of SARS-CoV-2 that was used in all the experiments was sequenced and deposited at GISAID (https://www.gisaid.org/) (accession number: EPI_ISL_1707039).

## References

[1] O’Sullivan ED (2020). PPE guidance for covid-19: Be honest about resource shortages. BMJ.

[2] Cohen J, Rodgers YVM (2020). Contributing factors to personal protective equipment shortages during the COVID-19 pandemic. Prev. Med..

[3] Salido RA (2020). Handwashing and detergent treatment greatly reduce SARS-CoV-2 viral load on Halloween candy handled by COVID-19 patients. mSystems.

[4] Biasin M (2021). UV-C irradiation is highly effective in inactivating SARS-CoV-2 replication. Sci. Rep..

[5] Leung A (2020). In vitro inactivation of SARS-CoV-2 using gamma radiation. Appl. Biosaf..

[6] Ibáñez-Cervantes G (2020). Disinfection of N95 masks artificially contaminated with SARS-CoV-2 and ESKAPE bacteria using hydrogen peroxide plasma: Impact on the reutilization of disposable devices. Am. J. Infect. Control..

[7] Ludwig-Begall LF (2020). The use of germicidal ultraviolet light, vaporized hydrogen peroxide and dry heat to decontaminate face masks and filtering respirators contaminated with a SARS-CoV-2 surrogate virus. J. Hosp. Infect..

[8] Ogilvie BH (2021). Alcohol-free hand sanitizer and other quaternary ammonium disinfectants quickly and effectively inactivate SARS-CoV-2. J. Hosp. Infect..

[9] Viscusi DJ (2009). Evaluation of five decontamination methods for filtering facepiece respirators. Ann. Occup. Hyg..

[10] Cadnum JL (2019). A comparison of the efficacy of multiple ultraviolet light room decontamination devices in a radiology procedure room. Infect. Control Hosp. Epidemiol..

[11] Guettari M, Gharbi I, Hamza S (2021). UVC disinfection robot. Environ. Sci. Pollut. Res..

[12] Storm N (2020). Rapid and complete inactivation of SARS-CoV-2 by ultraviolet-C irradiation. Sci. Rep..

[13] Sabino CP (2020). UV-C (254 nm) lethal doses for SARS-CoV-2. Photodiagn. Photodyn. Ther..

[14] Ma B (2021). UV inactivation of SARS-CoV-2 across the UVC spectrum: KrCl* excimer, mercury-vapor, and light-emitting-diode (LED) sources. Appl. Environ. Microbiol..

[15] Heßling M (2020). Ultraviolet irradiation doses for coronavirus inactivation—Review and analysis of coronavirus photoinactivation studies. GMS Hyg. Infect. Control..

[16] Reed LJ, Muench H (1938). A simple method of estimating fifty per cent endpoints. Am. J. Epidemiol..

[17] Bellon M (2021). Severe acute respiratory syndrome coronavirus 2 (SARS-CoV-2) viral load kinetics in symptomatic children, adolescents, and adults. Clin. Infect. Dis..

[18] Pujadas E (2020). SARS-CoV-2 viral load predicts COVID-19 mortality. Lancet Respir. Med..

[19] Pfeifer GP (1997). Formation and processing of UV photoproducts: Effects of DNA sequence and chromatin environment. Photochem. Photobiol..

[20] Beggs CB, Avital EJ (2020). Upper-room ultraviolet air disinfection might help to reduce COVID-19 transmission in buildings: A feasibility study. PeerJ.

[21] Martínez-Antón JC (2021). Determination of the characteristic inactivation fluence for SARS-CoV-2 under UV-C radiation considering light absorption in culture media. Sci. Rep..

[22] Woo MH (2012). Effects of relative humidity and spraying medium on UV decontamination of filters loaded with viral aerosols. Appl. Environ. Microbiol..

[23] Paton S (2021). Persistence of severe acute respiratory syndrome coronavirus 2 (SARS-CoV-2) virus and viral RNA in relation to surface type and contamination concentration. Appl. Environ. Microbiol..

[24] Gardner DWM, Shama G (1998). The kinetics of *Bacillus subtilis* spore inactivation on filter paper by u.v. light and u.v. light in combination with hydrogen peroxide. J. Appl. Microbiol..

[25] Gardner DW, Shama G (2000). Modeling UV-induced inactivation of microorganisms on surfaces. J. Food Prot..

[26] Fisher EM, Shaffer RE (2011). A method to determine the available UV-C dose for the decontamination of filtering facepiece respirators. J. Appl. Microbiol..

[27] Huber T (2021). Principles and practice for SARS-CoV-2 decontamination of N95 masks with UV-C. Biophys. J..

[28] Purschke M (2020). Construction and validation of UV-C decontamination cabinets for filtering facepiece respirators. Appl. Opt..

[29] Hong W (2021). Rapid determination of infectious SARS-CoV-2 in PCR-positive samples by SDS-PMA assisted RT-qPCR. Sci. Total Environ..

[30] Ashdown, I. *Modeling Spherical Irradiance for UV-C Air Disinfection. Consulted on the 28th of August 2022*. https://www.allthingslighting.org/?s=spherical+irradiance (2022).

[31] Kowalski, W. *Ultraviolet Germicidal Irradiation Handbook|SpringerLink. Consulted on the 28th of August 2022*. 10.1007/978-3-642-01999-9 (2009).

[32] Olsen M (2021). Mobile phones of paediatric hospital staff are never cleaned and commonly used in toilets with implications for healthcare nosocomial diseases. Sci. Rep..

