# Supplementary material for: Ultrafast inactivation of SARS-CoV-2 by 254-nm UV-C irradiation on porous and non-porous media of medical interest using an omnidirectional chamber
Source: Sci Rep. 2023 Aug 4;13:12648. doi: 10.1038/s41598-023-39439-1 (PMC10403608; doi:10.1038/s41598-023-39439-1)
Supplement: Supplementary file 1 — Supplementary Table S1. [file 41598_2023_39439_MOESM1_ESM.docx]

**Supplementary information**

Supplementary Table S1: Average Ct and Standard Deviation for disposable gowns, aprons, and horizontal and vertical stainless steel.
